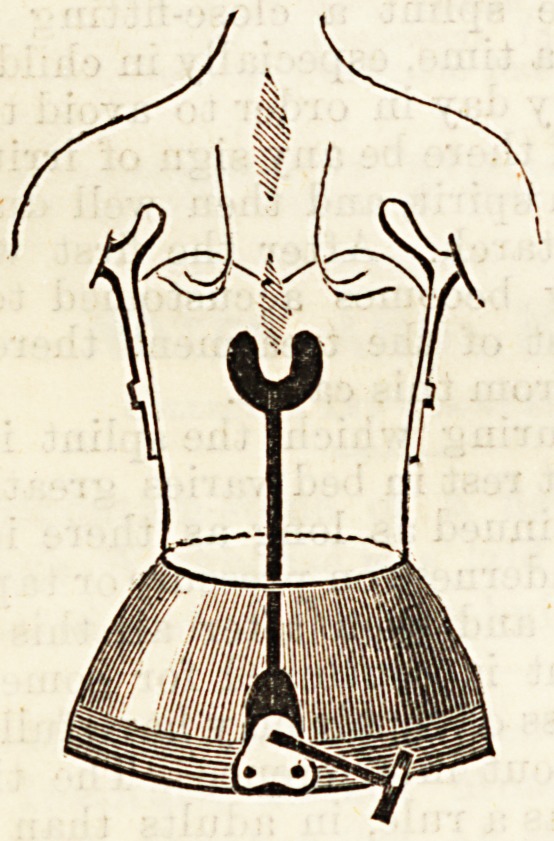# Caries of the Spine

**Published:** 1893-09-30

**Authors:** 


### The Hospital Clinic.

[*The Editor will be glad to receive offers of co-operation and contributions from members of the profession. All letters should be addressed to* The Editor, The Lodge, Porchester Square, London, W.]

### ROYAL ORTHOPÆDIC HOSPITAL.

### Caries of the Spine.

This disease is also known as angular curvature of the spine, because of the deformity that it causes, or as Pott's disease, in memory of Percival Pott, a celebrated surgeon of St. Bartholomew's Hospital, who first accurately described it in 1779. It is not possible fully to discuss its pathology here, yet in considering its treatment it is necessary to bear in mind that it is a true disease either of the vertebræ themselves or of the intervertebral discs. Hence it is an entirely different condition from lateral curvature of the spine which, at any rate in its early stages, is only an undue bending, and in some cases twisting, of the structures that make up the spinal column. Further, all these cases are inflammatory, and some, if not all, are tubercular. It is, therefore, not surprising that they show a tendency to form pus, which may ramify in various directions according to the point at which it is produced, but most commonly enters the sheath of the psoas muscle, and passing down inside it, appears at the upper part of the thigh as the well-known psoas abscess.

These cases are treated at the Royal Orthopædic Hospital by means of complete rest. This, which is regarded as the *sine quâ non*, is supplemented by good food, and by such medicinal treatment as each case seems to require. It is hoped that in this way the malnutrition that is always present, even in cases in which there is no absolute evidence of tubercular disease, may be overcome, and that the spine being at rest, the irritation caused by its continual movements, and by the weight of the body transmitted through it to the diseased part, may be removed, and thus the diseased part put under the most favourable conditions for being healed by the natural processes of repair.

Rest is ensured by means of a splint. This is made of thick leather which has been carefully moulded on a plaster cast of the back. It exactly corresponds to the shape of the spine, and accurately fits the deformed part. The splint is turned up at the sides so as to take hold of the body. Below, it comes down far enough to give firm support to the pelvis, and above, it stops just short of the head. In cases in which the cervical spine is diseased the splint is continued up so as to grasp the head, which is fastened to it by means of a band passing over the forehead. Two crutches of soft metal project forwards under the arms, and are bent upwards in front of the shoulders, so as to keep them at rest, and in contact with the splint. The body is fixed by means of a broad band of webbing, which passes over the chest and is laced up the front. The inside of the splint is padded and lined with soft leather. Experience has shown it to be a very efficient and comfortable apparatus, and the only objection that can be made to it is on the score of expense.

It was at one time thought that long-continued confinement to bed in the recumbent position would have a very serious effect on the health of the patient, and various forms of apparatus were tried in the hope that it might be possible to give support and ensure immobility to the spine, while at the same time permitting the patient to move about. The chief of these methods were the plaster of Paris jacket and Sayre's poroplastic jacket. These, however, have been entirely given up, as they have been found wanting as means of cure. It has further been shown that children confined to bed, even for long periods, suffer little, even if at all, provided their wants be properly attended to. In the case of adults the same cannot be said, and some of them find the confinement almost unbearable. It must, however, be borne in mind that angular curvature of the spine is a severe and often fatal disease, especially in adults, and that therefore it can hardly be expected that a cure can be wrought without trouble or discomfort. If a patient can be treated in pure country or seaside air, and can be daily taken out in a properly-constructed wheel chair, it is certain that a cure will more speedily be brought about. Unfortunately, however, these advantages cannot be obtained in the centre of London, and it is necessary to do the best that can be done without them.

Beneath the splint a close-fitting woollen vest is worn, and for a time, especially in children, the back is examined every day in order to avoid the risk of pressure sores. If there be any sign of irritation the place is bathed with spirit, and then well dusted with oxide of zinc and starch. After the first week or two the skin generally becomes accustomed to the pressure, and for the rest of the treatment there is usually no more trouble from this cause.

The time during which the splint is worn, and the patient kept at rest in bed varies greatly. The rule is that it be continued as long as there is any pain, discomfort, or tenderness on pressure or tapping about the diseased part, and even after all this has ceased the same treatment is continued for some weeks, in order that the process of repair may have full time to become complete without interference. The time required is much longer, as a rule, in adults than in children, in whom this period of recumbency generally ranges from about four to eight months. There is little advantage to be gained by unduly shortening it, while there is a risk of causing the formation of abscesses, and if it is necessary again to return to the recumbent treatment it is always found very troublesome and distressing to the patient.

A point of considerable practical importance in determining whether the wished-for bony anchylosis has taken place or not, is the disappearance of the puffiness which in the early stages of the disease is so marked around the projection, and the consequent apparent increase of the sharpness of the curve, owing to the fact that now for the first time its actual size can be accurately determined.

Abscesses are rarely seen except in cases that have come late under treatment. It is the custom at the Royal Orthopædic Hospital not to open them, but to aspirate as soon as they become tense and painful. Many dry up and disappear after having been aspirated several times.

When pain and tenderness has entirely disappeared, and has not returned even after a lapse of several weeks, the question of permitting the patient to get up arises. He is at first allowed to sit up in bed for a short time, and if no bad result follows the time is gradually extended, and at length he is permitted to be moved into a chair. If this can be done without harm, he is allowed to get up and move about. It is found advisable for the first few months that some means should be adopted in order to support the spine, which at first, as might be expected, feels very weak, and to prevent sudden jarring which might start the disease once more. For this purpose the instrument shown in the diagram on next page is ordered.

This instrument, which is moderately light considering its strength, is formed of a steel band surrounding the pelvis, from each side of which springs an upright reaching to the axilla, bearing on its upper end a crutch, which coming forward out of the armpit turns upwards in front of the shoulder. This crutch does not bear the weight of the body unless to a very small extent. Its main value is that it keeps the shoulders back, and that the upright supporting it gives attachment to a broad band of webbing, which, passing in front of the chest, and being laced up the middle line keeps the instrument in position. From the middle of the back of the pelvis band a strong steel upright rises. This is fixed with a rack joint, permitting it to move in an antero posterion plane. This upright carries a plate, shaped like a horse-shoe, which is covered with soft leather. This plate is of such shape and size that it closely surrounds all the projection of the spine, except its upper part, and while supporting it avoids the risk of sores that would arise if it pressed on the apex of the curve. By means of the joint before mentioned, the pressure is graduated to such an amount as is found desirable.

Another instrument is occasionally used by one of the surgeons, which has the advantages over the one just described that it is somewhat lighter, and a good deal cheaper. It is, however, only suitable for young patients, whose muscles have not very much strength. It simply consists of a soft pelvic band, to the middle of the back of which are attached two strips of soft flat iron. One of these runs up each side of the spinous processes of the vertebræ, and above they are attached to a band that passes forwards across the front of the chest, and is prevented from slipping down by leather straps over the shoulders.

It is obvious that the two instruments just described are only suitable for cases in which the deformity is in the dorsal, lumbo-dorsal, or lumbar regions. Since, however, the great majority of the cases are in these positions, it is only necessary to refer to treatment needed for the rarer cases of disease of the cervical spine. The only essential difference is that recumbency is insisted on for a longer period than in the other cases, that more caution is used in permitting the patient to get up, and that the instrument used on first walking about is carried up so as more or less to support the head.

Cases are sometimes met with in which, in addition to the usual angular curvature, there is a greater or less amount of lateral curvature. In these an instrument is applied which, in addition to the horse-shoe plate to support the angular projection, has also lateral plates which bear upon the lateral curves.

Occasionally, especially in cases that have been neglected during the early stages, paralysis gradually comes on, so that power of motion and, later, sensation may be entirely lost in the lower parts of the body and legs. Most of these cases entirely recover if kept at rest, although no special treatment be applied for this condition. Some of them, however, do not, and it is in these that laminectomy, or removal of the posterior laminæ of the vertebræ, has been so brilliantly successful in the hands of some surgeons. The effect of this operation is to give escape to inflammatory matters, and by removing the posterior wall of the rigid canal in which the spinal cord is contained, to allow of expansion of the contained tissues, and so free the cord from pressure. This operation has happily not been required at the Royal Orthopædic Hospital, at any rate within recent years. When the disease has been cured it is sometimes found that massage or electricity is of use in restoring power to the wasted muscles.

### Tubercular Disease of the Knee.

There is little difference between the treatment of this at the Royal Orthopædic Hospital and that in use in other institutions. The knee is kept at rest by means of some stiff apparatus, and then, when all inflammation has subsided, the attempt is made to restore its use by means of massage and careful manipulation. In some of the cases in which the knee is bent, it is found very advantageous to divide the flexor tendons before straightening the joint. It is found that the process of straightening is thus much shortened, there is much less pain, and the risk of displacing the tibia backwards is avoided.

## Figures and Tables

**Figure f1:**